# Alpha-synuclein fibrils induce budding of mitochondrial-derived vesicles

**DOI:** 10.1073/pnas.2604082123

**Published:** 2026-06-08

**Authors:** Thomas Braun, Viviane Reber, Cinzia Tiberi, Andri Fränkl, Dhiman Ghosh, Roland Riek, Tetiana Serdiuk

**Affiliations:** ^a^ Biozentrum, University of Basel, Basel 4056, Switzerland; ^b^Department of Biology, Institute of Molecular Systems Biology, Swiss Federal Institute of Technology Zürich, Zürich 8093, Switzerland; ^c^ BioEM Lab, Biozentrum, University of Basel, Basel 4058, Switzerland; ^d^ Department of Chemistry and Applied Biosciences, Institute of Molecular Physical Science, Swiss Federal Institute of Technology Zürich, Zürich 8093, Switzerland

## Abstract

α-synuclein (α-syn) aggregation is a hallmark of synucleinopathies, a class of neurodegenerative disorders such as Parkinson’s disease (PD). Several lines of evidence indicate the involvement of mitochondria in the disease pathology. Despite extensive study, the link between α-syn aggregation and mechanisms of mitochondrial toxicity remains not fully understood. Using high-resolution imaging with electron microscopy, we examined SH-SY5Y cells exposed to α-syn fibrils vs control cells with a focus on mitochondria. We found that upon exposure to α-syn fibrils, mitochondria cristae structure gets defects, and mitochondria enhance the budding of mitochondrial-derived vesicles (MDVs). MDV formation reflects an evolutionarily conserved mechanism reminiscent of bacterial outer membrane vesicle biogenesis. Structural proteomics analysis by mass spectrometry corroborates this microscopy observation by identifying changes in multiple proteins that regulate cristae structure, MDV formation, and trafficking. Our results suggest that α-syn may promote MDV generation, and support an important link between α-syn and mitochondria which will be important for future mechanistic studies. The processes we detected could be of interest for diagnostics and potential therapeutic interventions.

Both α-synuclein (α-syn) and mitochondria contribute to multiple neurodegenerative diseases collectively called synucleinopathies. The aggregation and pathological accumulation of α-syn in Lewy bodies are hallmarks of Parkinson’s disease (PD), where mitochondrial dysfunction is seen as both a contributor to and a consequence of α-syn pathology (1, 2). Furthermore, environmental factors such as toxins like rotenone and MPTP induce PD-like pathology by impairing mitochondrial respiratory chain function, promoting oxidative stress, and triggering α-syn aggregation (3–5).

Ultrastructural analyses of Lewy bodies in PD brains revealed that these inclusions are often rimmed with damaged mitochondria (6). Furthermore, α-syn has shown a conformation-dependent influence on mitochondrial dynamics and function (7), including ATP synthesis (8, 9), and transport along microtubules (10). Still, how α-syn fibrils impact mitochondria and the crosstalk between α-syn and mitochondria in pathology remain poorly understood.

Mitochondria are large organelles whose structural defects can be directly visualized by electron microscopy (EM). Here, we set out to characterize how mitochondrial morphology changes in response to a cell’s internalization of α-syn fibrils and how mitochondrial proteomes respond to fibrillar invasion. We found that internalization of α-syn fibrils caused cristae defects and enhanced mitochondrial-derived vesicle (MDVs) budding, linking α-syn toxicity to a recently discovered mitochondrial quality control system-MDV pathway.

## Results

**Fig. 1.**
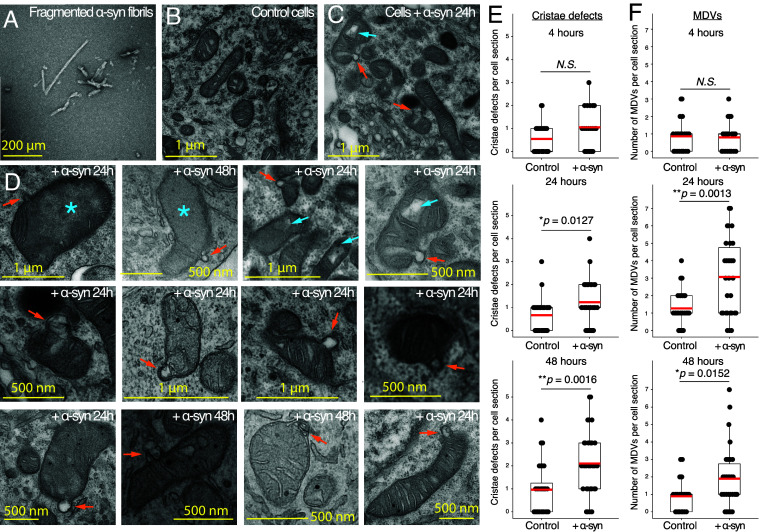
α-syn fibrils induce time-dependent mitochondrial defects. (*A*) EM image of fragmented α-syn fibrils used for seeding. (*B* and *C*) EM images of mitochondria in control SH-SY5Y cells (*B*) and SH-SY5Y cells treated with α-syn fibrils (250 nM monomer equivalents) for 24 h (*C*). (*D*) EM images illustrating mitochondrial cristae defects and budding of MDVs in cells treated with α-syn fibrils. (*E*) Quantification of the number of mitochondria with cristae defects per cell section in control and α-syn–treated cells at the indicated incubation times. (*F*) Quantification of MDVs per cell section in control and α-syn–treated cells. Black dots represent individual cells, and red lines indicate the mean. Statistical significance was assessed using two-tailed Wilcoxon rank-sum tests (**P < 0.01, *P < 0.05; *N.S*., not significant). For each condition (treated and control), *n* = 80 cells were analyzed in total across all incubation times. Cristae defects are labeled in blue: arrows indicate abnormal cristae morphology, and asterisks (*) indicate the absence of cristae structures. Orange arrows indicate MDVs.

We studied how α-syn fibrils impact mitochondria morphology using the well-established endocytic uptake of α-syn fibrillar fragments as a model. In short, we used fibrillar aggregates of purified full-length, N-terminally acetylated human α-syn, which were fragmented to fibril lengths of approximately 50 nm. We exposed SH-SY5Y cells to the α-syn fibrillar fragments (250 nM monomer equivalent concentration, Fig. 1*A*), for 4 h, 24 h, and 48 h washed them with cell culture media two times, and fixed them with a solution of 2% paraformaldehyde and 2.5% glutaraldehyde afterward. Fixed cells were resin-embedded, cut into slices, and imaged by EM (Methods). At least 80 cells were analyzed per condition (treated and control, across different incubation times) from different preparations.

We have analyzed EM data on several different axes regarding mitochondrial morphology: cristae shape and budding of MDVs. A representative overview of nonseeded (B) and α-syn seeded (C) cells is given in Fig. 1. Representative “zoom-in” images of mitochondria showing different morphological aberrations upon α-syn treatment are shown in Fig. 1*D*. While inspecting cells treated with α-syn fibrils, we found that mitochondrial cristae significantly more often exhibited peculiar defects at 24 and 48 h, but not at 4 h (Fig. 1*E*). These defects included atypical cristae shapes (e.g., trapezoidal) or partial to complete loss of cristae structure. Often, mitochondria depleted of cristae structure were also obviously larger than the other mitochondria. Close inspection of mitochondria after treatment with α-syn fibrils revealed significant differences in membrane budding at 24 and 48 h, but not at 4 h (Fig. 1*F*). Several examples of mitochondria budding MDVs are shown in Fig. 1*D*. MDVs are one of the pathways of mitochondrial quality control used by a cell to remove damaged material from mitochondria when mitophagy is still avoidable. We have seen approximately two times more MDVs on the EM images of cells treated with α-syn fibrils than the control cells treated with the same volume of PBS at prolonged incubation times (24 h and 48 h, Fig. 1*F*). This result could indicate the response to enhanced mitochondrial stress caused by α-syn fibrils.

**Fig. 2.**
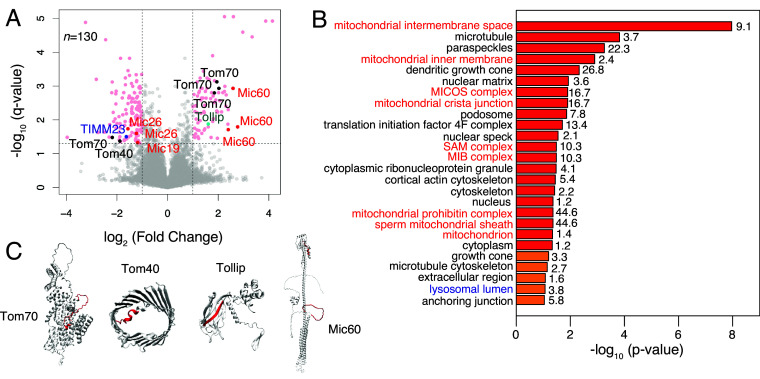
α-syn fibrils affect the mitochondrial structural proteome of SH-SY5Y cells. (*A*) Volcano plot of proteins showing structural change due to uptake of α-syn fibrils. Significance cut-off of *q* < 0.05 and FC > 2 was used. (*B*) Gene Ontology (Cellular Component) enrichment analysis of LiP-MS hits. Red bars indicate terms enriched at *P* < 0.05, and orange bars indicate terms enriched at *P* < 0.1. Numbers adjacent to each bar show the fold enrichment, calculated as the ratio of the observed term frequency in LiP-MS hits to the expected frequency based on the detected proteome. (*C*) Examples of LiP-MS hits with changing peptides mapped on the solved or predicted structures for TOM70 (PDB ID AF-O94826-F1), TOM40 (PDBID 7ck6), Tollip (AF-Q9H0E2), and MIC60 (AF-Q16891-F1).

To support our visual results on the molecular level, we performed an unbiased structural proteomics analysis with Limited proteolysis coupled to mass spectrometry (LiP-MS). LiP-MS probes proteome accessibility to sequence unspecific protease, proteinase K (PK), across different conditions. LiP-MS can detect variable structural changes, including changes in protein–protein interactions, enzymatic activity, aggregation, conformational changes, and posttranslational modifications (11). We applied LiP-MS to cells treated with α-syn fibrils and control cells treated with PBS and found 130 proteins structurally altered upon treatment with α-syn, reflecting direct and functional interactions (Fig. 2*A*). Gene Ontology enrichment analysis revealed that multiple cellular compartments were enriched (Fig. 2*B*), with mitochondria-related terms among the most prominent, including the mitochondrial inner membrane and intermembrane space, the MICOS complex, the prohibitin complex, and MIB complexes. Multiple of these mitochondrial proteins are important for mitochondrial cristae organization. For instance, several proteins of the MICOS complex (Mic60, Mic19, Mic26) were among the strongest screening hits. The outer membrane translocase elements TOM40 and TOM70 and the inner membrane translocase TIMM23 changed the PK susceptibility upon α-syn treatment (Fig. 2*A*). All these proteins are crucial for mitochondria cristae structure, mitochondrial biogenesis, maintenance of contact sites between inner and outer mitochondrial membrane, and mitochondria membrane remodeling. LiP-MS allowed us to identify the regions of structural alteration on these proteins (Fig. 2*C*). Remarkably, the Tollip protein, shown to be essential for TOM complex-positive MDV trafficking, along with the TOM complex, structurally responded to the α-syn fibrils treatment, corroborating our EM data on enhanced MDVs formation (12). Given its important function in maintaining mitochondria architecture, the MICOS complex was also suggested to play a role in the formation of MDVs (13), possibly interacting with Rho GTPases MIRO1/MIRO2. Interestingly, along with detected structural changes in many subunits of the MICOS complex, we also detected a moderate structural change for MIRO2 (FC>1.5, *P* <0.05).

## Discussion

Mitochondrial quality control relies on several mechanisms, including mitochondrial proteases, mitophagy, and MDVs (14). Whereas the role of mitophagy in PD is well understood, with established genetic links (PINK1, Parkin), MDVs represent a more recently described quality control pathway complementing mitophagy. MDVs selectively deliver damaged mitochondrial components (proteins and lipids) to lysosomes or peroxisomes for degradation. However, the mechanisms regulating MDV biogenesis, their functional impact on mitochondrial homeostasis, and their intracellular fate remain incompletely understood and represent important directions for future work. We observed increased vesicular structures consistent with MDVs upon cell treatment with α-syn fibril fragments. These vesicles displayed hallmarks of previously described MDVs, including sizes of ~70 to 150 nm, absence of cristae, and apparent budding from the outer mitochondrial membrane, distinguishing them from larger fragments retaining cristae (14).

This effect was time-dependent: No significant change was observed after short incubation times (4 h), whereas MDV generation was significantly induced at longer incubation times (24 and 48 h). We did not extend the incubation time beyond 48 h because the SH-SY5Y cells used here are dividing and express low levels of endogenous α-syn. Interestingly, the mitochondria of the cells treated with α-syn fibrils had more cristae defects than the control cells. Some large mitochondria were almost completely depleted of cristae (Fig. 1*D*). Whether α-syn fibrils are incorporated into MDVs or trigger their formation through external membrane interactions remains to be determined. Future studies using immunogold electron microscopy and subcellular fractionation approaches could clarify the spatial relationship between α-syn fibrils and MDVs. Additionally, combining advanced live-cell imaging with correlative EM could provide further insight into the impact of α-syn fibrils on mitochondrial morphology.

In support of our microscopy data, we have previously shown that α-syn fibrils impair ATP production (15). Structural proteomics further revealed changes in multiple proteins involved in the MDV pathway in response to cellular treatment with α-syn. For these and other LiP-MS mitochondrial protein hits, we mapped regions of structural alteration with a resolution of approximately 10 amino acids (Fig. 2*C*).

The enhanced release of MDVs was recently described in murine brains with Down Syndrome (16), and the findings were confirmed in human postmortem brain material. It has been suggested that MDV release is enhanced in Alzheimer’s disease and neurodegeneration in general (17) and that MDVs are released extracellularly (16, 17). Therefore, MDVs are potentially interesting for early diagnostics. Here, we showed that α-syn fibrils can directly trigger enhancement of MDV release, establishing the link between α-syn aggregation and mitochondria stress. It remains to be evaluated whether tau and amyloid-β fibrils also can directly cause the enhancement of MDV formation.

## Methods

N-terminally acetylated α-syn was purified as described previously (18). EM images were analyzed using Fiji. Two-tailed Wilcoxon rank-sum test was used to assess statistical significance. LiP-MS experiments and analysis were performed as described previously (11). Extended methods can be found in *SI Appendix*.

## Supplementary Material

Appendix 01 (PDF)

## Data Availability

Proteomics data have been deposited in PRIDE with the dataset identifier PXD078690 (19).
