# Supplementary material for: Alpha-synuclein fibrils induce budding of mitochondrial-derived vesicles
Source: Proc Natl Acad Sci U S A. 2026 Jun 8;123(24):e2604082123. doi: 10.1073/pnas.2604082123 (PMC13273258; doi:10.1073/pnas.2604082123)
Supplement: Supplementary file 1 — Appendix 01 (PDF) [file pnas.2604082123.sapp.pdf]

Methods

Cell seeding

SH-SY5Y neuroblastoma cells (ATCC) were cultured in DMEM/F-12 GlutaMAX medium (#10565018, Thermo Fisher Scientific) supplemented with 10% fetal bovine serum (FBS) and 1% penicillin/streptomycin. For treatment, cells were exposed to 250 nM α-synuclein (α-syn) fibrils (monomer equivalent) in complete culture medium for 4 h, 24 h, or 48 h. PBS-treated control cells were cultured in parallel under identical conditions. Two independent α-syn fibril preparations were used for the 24 h and 48 h time points, and a single preparation was used for the 4 h time point. For the 24 h and 48 h experiments, the experiments were repeated using independently thawed and cultured cells to ensure reproducibility.

Electron microscopy

SH-SY5Y cells treated with α-syn fibrils or mock cells were fixed using a solution containing 2% paraformaldehyde (PFA) and 2.5% glutaraldehyde (GA) in 0.1M PIPES buffer. Following fixation, the cells were impregnated in 2% low-melting-point agarose and placed on ice for 20 minutes to allow the agarose to solidify. The resulting solid agarose pellets were carefully removed from the tubes and trimmed into smaller pieces using a razor blade.

Agarose cubes containing sample material were transferred to small glass vials, thoroughly washed with 0.1 M Cacodylate buffer, and post-fixed in 1% buffered osmium tetroxide for 1 hour at 4 °C. Samples were then rinsed with distilled water and stained *en bloc* with aqueous uranyl acetate for 1 hour at 4 °C in the dark. After staining, samples were dehydrated through a graded ethanol series. After three changes of absolute ethanol, samples are washed in acetone and finally embedded in a mixture of resin/acetone first and then in pure Epon 812 resin. Embedding is carried out in a 60°C oven for 48 h until the epoxy resin is completely hardened and ready to section.

Ultrathin sections (70 nm thick) were cut using an ultramicrotome (Leica EM UC7, Leica Microsystems, Austria) and contrasted with uranyl acetate and lead citrate. The sections were imaged using a FEI Tecnai G2 Spirit transmission electron microscope (TEM) operated at 80 kV. Images were acquired with anEMSIS Veleta digital camera (top, side-mounted) using RADIUS software (EMSIS GmbH). EM images were analyzed using Fiji.(1)

The number of MDVs per cell profile in ultrathin sections was quantified by manual counting. Cristae defects were defined as mitochondria displaying abnormal cristae morphology, including altered shape (e.g., trapezoidal geometry) or partial or complete loss of cristae structure. In analogy to MDV quantification, the number of mitochondria exhibiting cristae defects was determined per cell section. The observed increase in MDVs and cristae defects was robust to the normalization strategy. Similar results were obtained when values were expressed per cell section or normalized to the number of mitochondria.

LiP-MS

Proteomics analysis

Native cell lysis

Pellets of SH-SY5Y cells were resuspended in 200 µl of ice-cold LiP buffer (100 mM HEPES, 150 mM KCl, 1 mM $MgCl_2$, pH 7.4). To lyse cells, we performed 10 cycles of 10 douncing steps using a pellet pestle on ice. A bicinchoninic acid (BCA) assay was used to measure protein concentrations. After native lysis and concentration determination, the samples were processed directly by LiP-MS.

LiP-MS

The concentrations of cell lysates were generally similar between replicates, with minor differences adjusted with LiP buffer (20 mM HEPES, 150 mM KCl, 1 mM $MgCl_2$, at pH 7.4). All samples were diluted in LiP buffer to a final protein concentration of 2mg/ml and a total volume of 50 µl per sample. Six replicates per each condition were used.

LiP-MS experiments were conducted as described previously with some minor modifications(2-4). Briefly, 5 µl of Proteinase K (PK) solution was added at an enzyme-to-substrate ratio of 1:100 (w/w) using a multichannel pipette. Samples were briefly mixed by gentle pipetting up and down 5 times. Next, the samples were incubated at 37°C for exactly 5 min in a Biometra TRIO thermocycler. PK digestion of proteomes was stopped by incubating the samples at 99°C for 5 min, followed by a 5 min incubation on ice. Subsequently, 55 µl of freshly prepared 10% sodium deoxycholate (DOC) was added to each sample.

Trypsin / LysC digestion

Samples were reduced with tris(2-carboxyethyl)phosphine hydrochloride (TCEP) to a final concentration of 5 mM at 37 °C for 40 minutes with shaking at 800 rpm in a thermomixer (Eppendorf). Following reduction, samples were alkylated by adding iodoacetamide (IAA) to a final concentration of 40 mM and incubated for 30 minutes at room temperature in the dark. To reduce the concentration of sodium deoxycholate (DOC) to 1%, samples were diluted with 100 mM ammonium bicarbonate (Ambic). Proteolytic digestion was carried out by adding Lysyl endopeptidase (LysC, Wako Chemicals) and sequencing-grade trypsin (Promega) at an enzyme-to-substrate ratio of 1:100. Digestion was performed overnight at 37 °C in a thermomixer with continuous shaking at 800 rpm. The reaction was stopped by adding formic acid (FA, Carl Roth GmbH) to a final concentration of 3%, resulting in DOC precipitation. DOC was removed by three rounds of centrifugation at 21,000 rcf for 20 minutes each, with the supernatant collected after each spin. The combined supernatants were desalted using Sep-Pak tC18 cartridges (Waters) and eluted with 80% acetonitrile containing 0.1% formic acid.

LC-MS/MS data acquisition

Liquid chromatography

LiP-MS samples were analyzed using an Orbitrap Exploris mass spectrometer (Thermo Fisher Scientific) coupled to a nano-electrospray ion source and a nano-flow LC system (Easy-nLC 1200, Thermo Fisher Scientific). Peptide separation was performed using self-packed 40 cm x 0.75 mm columns (New Objective) containing 1.9 µm C18 beads. A linear gradient of LC buffer A (5 % ACN, 0.1 % FA, Carl Roth GmbH) and LC buffer B (95 % ACN, 0.1 % FA, Carl Roth GmbH) increasing from 3 % to 30 % of LC buffer B over 120 minutes at a flow rate of 300 nl/minute was used.

Data-independent acquisition

Data-independent acquisition (DIA)(5) scans were performed using 41 variable-width isolation windows. Precursor ions were isolated with a quadrupole. For MS1 survey scans, a mass range of 350-1150 m/z was used and an Orbitrap resolution of 120’000. A normalized automated gain control (AGC) target of 200 % was applied. High-energy

collision induced dissociation (HCD) was employed to fragment precursor ions. DIA-MS/MS spectra were recorded in the Orbitrap with a resolution of 30’000 and a mass range of 350-1150 m/z. The maximal injection time was set to 66 ms.

Data-dependent acquisition

Samples were acquired in Data-dependent mode (DDA) alongside DIA runs to generate spectral libraries. Precursor ions were isolated using a quadrupole. For MS1 survey scans, a mass range of 350-1150 m/z was used. Precursor ions with intensities above 5’000 were selected for MS/MS scans. The isolation window of the quadrupole was 1.4 m/z. HCD was employed to fragment precursor ions. MS/MS spectra were acquired on an Orbitrap at a resolution of 30’000, an AGC target of 200% and a maximum injection time of 54 ms.

Data search, statistical data analysis and visualisation

Spectral libraries were generated through a Pulsar search in Spectronaut (Biognosys AG). The raw DIA MS data were also searched in Spectronaut using generated libraries. Compared to the default settings, the specificity was adjusted to semi-specific for Trypsin/P. This adjustment accounts for semi-tryptic peptides generated by PK. LiP-MS data were analysed at the peptide level. To compare peptide abundance, we performed a two-tailed Welch t-test with Benjamini-Hochberg correction after median normalization. p-Values adjusted for multiple comparisons were referred to as q-values. Protein group quantities were used for protein abundance comparison. The significance threshold FC > 2, q-val < 0.05 was applied. The statistical analysis was conducted in R using the protti and limma packages. For normalization, peptide intensities were divided by the corresponding control protein abundance per peptide before comparing peptide abundances. Protein abundance was assessed in the same lysates that had been digested with trypsin only in parallel to the LiP-MS workflow. The same sample processing protocol was followed for tryptic control samples, except adding PK. GO enrichment analysis was carried out using David(6, 7). The identified protein set served as the background for GO Enrichment analysis. LiP-MS hits were visualized and mapped on protein structures using Visual Molecular Dynamics (VMD).

References

1. J. Schindelin, I. Arganda-Carreras, E. Frise, V. Kaynig, M. Longair, T. Pietzsch, S. Preibisch, C. Rueden, S. Saalfeld, B. Schmid *et al.*, Fiji: an open-source platform for biological-image analysis. *Nat Methods* **9**, 676-682 (2012).
2. P. Stalder, T. Serdiuk, D. Ghosh, Y. Fleischmann, N. Ait-Bouziad, J. P. Quast, L. Malinovska, A. Ouared, A. Davranche, W. Haenseler *et al.*, An approach to characterize mechanisms of action of anti-amyloidogenic compounds in vitro and in situ. *NPJ Parkinsons Dis* **11**, 122 (2025).
3. A. Holfeld, D. Schuster, F. Sesterhenn, A. K. Gillingham, P. Stalder, W. Haenseler, I. Barrio-Hernandez, D. Ghosh, J. Vowles, S. A. Cowley *et al.*, Systematic identification of structure-specific protein-protein interactions. *Mol Syst Biol* **20**, 651-675 (2024).
4. S. Schopper, A. Kahraman, P. Leuenberger, Y. Feng, I. Piazza, O. Muller, P. J. Boersema, P. Picotti, Measuring protein structural changes on a proteome-wide scale using limited proteolysis-coupled mass spectrometry. *Nat Protoc* **12**, 2391-2410 (2017).
5. L. C. Gillet, P. Navarro, S. Tate, H. Rost, N. Selevsek, L. Reiter, R. Bonner, R. Aebersold, Targeted data extraction of the MS/MS spectra generated by data-independent acquisition: a new concept for consistent and accurate proteome analysis. *Mol Cell Proteomics* **11**, O111 016717 (2012).
6. W. Huang da, B. T. Sherman, R. A. Lempicki, Systematic and integrative analysis of large gene lists using DAVID bioinformatics resources. *Nat Protoc* **4**, 44-57 (2009).
7. B. T. Sherman, M. Hao, J. Qiu, X. Jiao, M. W. Baseler, H. C. Lane, T. Imamichi, W. Chang, DAVID: a web server for functional enrichment analysis and functional annotation of gene lists (2021 update). *Nucleic Acids Res* **50**, W216-W221 (2022).
